# Supplementary material for: The chronic kidney disease and acute kidney injury involvement in COVID-19 pandemic: A systematic review and meta-analysis
Source: PLoS One. 2021 Jan 5;16(1):e0244779. doi: 10.1371/journal.pone.0244779 (PMC7785235; doi:10.1371/journal.pone.0244779)
Supplement: Attachment — Submitted filename: response to reviewers.docx [file pone.0244779.s004.docx]

Dear editor,

Thank you for your letter and for the comments concerning our manuscript entitled “The Chronic Kidney Disease and Acute Kidney Injury Involvement in COVID-19 Pandemic: A Systematic Review and Meta-analysis” (PONE-D-20-22031). Those comments are all of great value for revising and improving our paper. We have studied the comments carefully and have made modifications which we hope can meet with approval. The responses to the reviewer’s comments are as follows:

**Comments to the Author**
1. Is the manuscript technically sound, and do the data support the conclusions?
Response: The manuscript is technically sound, and the the data supports the conclusions.
The manuscript must describe a technically sound piece of scientific research with data that supports the conclusions. Experiments must have been conducted rigorously, with appropriate controls, replication, and sample sizes. The conclusions must be drawn appropriately based on the data presented.

Reviewer #1: Yes

Reviewer #2: No

2. Has the statistical analysis been performed appropriately and rigorously?

Response: The statistical analysis has been performed appropriately and rigorously based on Review Manager software 5.3.

Reviewer #1: I Don't Know

Reviewer #2: I Don't Know

3. Have the authors made all data underlying the findings in their manuscript fully available?
Response: We have made all data underlying the findings in their manuscript fully available.
The [PLOS Data policy](http://www.plosone.org/static/policies.action#sharing) requires authors to make all data underlying the findings described in their manuscript fully available without restriction, with rare exception (please refer to the Data Availability Statement in the manuscript PDF file). The data should be provided as part of the manuscript or its supporting information, or deposited to a public repository. For example, in addition to summary statistics, the data points behind means, medians and variance measures should be available. If there are restrictions on publicly sharing data—e.g. participant privacy or use of data from a third party—those must be specified.

Reviewer #1: Yes

Reviewer #2: Yes

4. Is the manuscript presented in an intelligible fashion and written in standard English?
Response: The manuscript is presented in an intelligible fashion and written in standard English.
PLOS ONE does not copyedit accepted manuscripts, so the language in submitted articles must be clear, correct, and unambiguous. Any typographical or grammatical errors should be corrected at revision, so please note any specific errors here.

Reviewer #1: Yes

Reviewer #2: No

5. Review Comments to the Author

Please use the space provided to explain your answers to the questions above. You may also include additional comments for the author, including concerns about dual publication, research ethics, or publication ethics. (Please upload your review as an attachment if it exceeds 20,000 characters)

Reviewer #1: Authors conducted a systematic review & meta-analysis of risk of kidney involvement according the severity of COVID19. Although the objective of the manuscript is interesting enough and deserves publication, the current version of the manuscript is incomplete in terms of quality according to PRISMA guideline and needs revisions according to the comments below.

1. First paragraph of the Introduction should be more concise and to the point.

Response: According to your suggestion, we have revised the first paragraph of the Introduction.

2. "to verify whether kidney diseases are involved in COVID19" is very vague. Objective of the study should be more clearly defined (authors seems to only assess increasing prevalence of CKD or AKi according to the severity of COVID19).

Response: We have adjusted it as “to verify whether preexisting CKD is a risk factor for severe COVID-19, and whether AKI is a complication for severe COVID-19.”
3. Authors should provide SR registration number as in PROSPERO.

Response: We were trying to register this systematic review in PROSPERO. However, we gave up because of time urgency, and we published it in medRxiv in May 02, 2020 to attract more attention for the potential kindey injury. Therefore, we missed the opportunity to register it in PROSPERO.
4. In selecting the studies, AKI or CKD should be clearly defined, which seems vague in this study.

Response: AKI was clearly defined in six included studies. In five included studies, AKI was defined according to the Kidney Disease: Improving Global Outcomes (KDIGO) criteria. In addition, AKI was defined as any of renal functional indices beyond normal range in one included study. Unfortunately, none of the included studies mentioned the CKD diagnostic criteria probably because CKD is a preexisting disease. AKI is a complication of COVID-19, which probably cause most of the included studies did not mention its diagnostic criteria. Considering these reasons, AKI and CKD were not clearly defined in this study.

5. Studies selected are only from China, but those from other area should be included for applicability of the study results.

Response: As indicated in inclusion criteria, the publcation area and language were not limited in this systematic review. We were planning to include studies from all over the world to increase the credibility of results. However, all the included studies are only from China, which restrict its applicability in other areas.
6. Authors should provide at least one literature search formula, so that readers/reviewers can reproduce the study results.

Response: The literature search formula were as follws:

For English databases:

#1“SARS-CoV-2” (all fields or all text)

#2 “2019-nCoV” (all fields or all text)

#3 “COVID-19” (all fields or all text)

#4“novel coronavirus pneumonia” (all fields or all text)

#5 (#1 OR #2 OR #3 OR #4)

#6 “kidney” (all fields or all text)

#7 “renal” (all fields or all text)

#8 (#6 OR #7)

#9 (#5 AND #8)

For Chinese databases:

#1 “xin xing guan zhuang bing du” (subject)

#2 “xin xing guan Zhuang bing du fei yan” (subject)

#3 (#1 OR #2)
7. Risk of Bias table should be presented. For NOS score, only the total score is not enough but needs to present which item acquired the score in each selected study.

Response: With your suggestion, the detailed NOS score have be provided in the supplementary materials.
8. Last paragraph of the Discussion should not be the recommendation. Recommendation should be derived from the current study, but it did not provide any information for the recommended items.

Response: According to your suggestion, we have deleted the recommendation part.
9. Conclusion should be confined to the one derived from the results gained in this study. Need for CRRT cannot be concluded from the study.

Response: The result showed that severe group had higher ratio of receiving CRRT than nonsevere group (*P* < 0.00001, OR = 23.63, 95% CI: 8.34 to 66.95). It is concluded that CRRT is applied more frequently in the severe group than that in the nonsevere group. However, need for CRRT were not concluded from the study. We speculate that there is some kind of misunderstanding.

Reviewer #2: This review article evaluated involvement of CKD and AKI in COVID-19 patients. Due to the high impact of COVID-19 on public healthcare all over the world, I believe there is useful information in this manuscript. However, there are many inaccurate wordings in the current manuscript, which make it difficult to understand the appropriate conclusions from this study. Overall, I believe what the authors found were 1. CKD is a risk factor of severe COVID-19, and 2. Higher complication rates of AKI were found in severe COVID-19 patients when compared to non-severe cases. This is something already reported, but additional publications should be welcomed by researchers. Followings are my major comments.

1. The definition of severe or non-severe COVID-19 cases is unclear. Because of this, it was not possible to evaluate the manuscript appropriately. Does the severe case mean patients who required RRT, intubation, or something else?

Response: With in-depth understanding of COVID-19, the severity classification for COVID-19 is also changing according to China national diagnosis and treatment guidelines. In some earlier studies, patients were divided into two groups: severe group and nonsevere group. Subsequently, patients were divided into multiple groups, including mild, moderate, severe, and critical in later studies. Therefore, we divided all the included studies into two subgroups: severe group compared with nonsevere group, critical group compared with severe group. Severe COVID-19 is defined as respiratory rate ≥ 30 times/min, or oxygen saturation ≤ 93 % during resting state, or PaO_2_ / FiO_2_ ≤ 300 mmhg, or progress in imaging of lung lesions > 50%.

2. In the introduction, the number of COVID patient is outdated. Please update.

Response: With your suggestion, we have updated the number of COVID-19 patient.
3. At the end of introduction, authors said “to verify whether kidney diseases are involved in COVID-19”. This is an unclear statement. If the purpose of this study is to determine whether preexisting CKD is a risk factor for severe COVID19, then authors should explain so. If the purpose was to evaluate whether AKI is a complication in severe COVID19 cases, that should be also explained.

Response: Thank you very much for your kind reminder. Under your suggestion, we have revised the last sentence of the Introduction.
4. In study description, authors explained “All the included studies were performed in China”. What is the reason why studies from outside China were excluded?

Response: We were planning to include enough studies all over the world to lower region bias and increase the quality of evidence. However, the studies from outside China were excluded because of unavailable outcomes.
5. Due to unclear wording, it is difficult to understand the results. For example, in Comorbidity of CKD, authors said “the comorbidity of CKD was significantly increased in the severe group than that in the nonsevere groups.”. This sounds like the CKD was developed more frequently in severe group after COVID19 infection. But, I believe what authors meant was the patients with CKD were more likely to develop severe COVID19. The same is true to serum creatinine, blood urea nitrogen, and etc.

Response: Thank for your understanding. We have adjusted it as “propotion of preexisting CKD was significantly increased in the severe group than that in the nonsevere group”. In terms of serum creatinine, blood urea nitrogen and other outcomes, they are not preexisted.
6. In the result section, “serum creatinine” and “blood urea nitrogen” were repeated twice. This is confusing and needs to be corrected.

Response: “Serum creatinine” and “blood urea nitrogen” were compared in two differet subgroups: severe group compared with nonsevere group, critical group compared with severe group. In each subgroup, “serum creatinine” and “blood urea nitrogen” refer to specific values. Meanwhile, “abnormal serum creatinine” and “abnormal blood urea nitrogen” refer to the number of patients with elevated serum creatinine or blood urea nitrogen.
7. In discussion, authors claimed this is the first research to evaluate kidney involvement in COVID-19 patients. There are many papers published. This is probably overclaim and needs to be revised.

Response: We published the article in medRxiv in May 02, 2020. Afterwards, many similar papers published. Anyway, we are glad to revise the sentence.

We would like to express our great appreciation to you. Looking forward to hearing from you.

Yours sincerely,

Ya-Fei Liu
